# Butin (7,3′,4′-Trihydroxydihydroflavone) Reduces Oxidative Stress-Induced Cell Death via Inhibition of the Mitochondria-Dependent Apoptotic Pathway

**DOI:** 10.3390/ijms12063871

**Published:** 2011-06-10

**Authors:** Rui Zhang, In Kyung Lee, Mei Jing Piao, Ki Cheon Kim, Areum Daseul Kim, Hye Sun Kim, Sungwook Chae, Hee Sun Kim, Jin Won Hyun

**Affiliations:** 1 School of Medicine and Applied Radiological Science Research Institute, Jeju National University, Jeju-si 690-756, Korea; E-Mails: zhangrui26@hotmail.com (R.Z.); meijing0219@hotmail.com (M.J.P.); svv771@hotmail.com (K.C.K.); candy4860@hanmail.net (A.D.K.); 2 Cancer Research Institute, Seoul National University College of Medicine, Seoul 110-799, Korea; E-Mails: inkyeong@korea.ac.kr (I.K.L.); hyisun@snu.ac.kr (H.S.K.); 3 Aging Research Center, Korea Institute of Oriental Medicine, Daejeon 305–811, Korea; E-Mail: cdcl3@naver.com; 4 Department of Neuroscience, College of Medicine, Ewha Womans University, Seoul 110-783, Korea; E-Mail: hskimp@ewha.ac.kr

**Keywords:** butin, oxidative stress, mitochondria-dependent apoptotic pathway

## Abstract

Recently, we demonstrated that butin (7,3′,4′-trihydroxydihydroflavone) protected cells against hydrogen peroxide (H_2_O_2_)-induced apoptosis by: (1) scavenging reactive oxygen species (ROS), activating antioxidant enzymes such superoxide dismutase and catalase; (2) decreasing oxidative stress-induced 8-hydroxy-2′-deoxyguanosine levels via activation of oxoguanine glycosylase 1, and (3), reducing oxidative stress-induced mitochondrial dysfunction. The objective of this study was to determine the cytoprotective effects of butin on oxidative stress-induced mitochondria-dependent apoptosis, and possible mechanisms involved. Butin significantly reduced H_2_O_2_-induced loss of mitochondrial membrane potential as determined by confocal image analysis and flow cytometry, alterations in Bcl-2 family proteins such as decrease in Bcl-2 expression and increase in Bax and phospho Bcl-2 expression, release of cytochrome c from mitochondria into the cytosol and activation of caspases 9 and 3. Furthermore, the anti-apoptotic effect of butin was exerted via inhibition of mitogen-activated protein kinase kinase-4, c-Jun NH_2_-terminal kinase (JNK) and activator protein-1 cascades induced by H_2_O_2_ treatment. Finally, butin exhibited protective effects against H_2_O_2_-induced apoptosis, as demonstrated by decreased apoptotic bodies, sub-G_1_ hypodiploid cells and DNA fragmentation. Taken together, the protective effects of butin against H_2_O_2_-induced apoptosis were exerted via blockade of membrane potential depolarization, inhibition of the JNK pathway and mitochondria-involved caspase-dependent apoptotic pathway.

## 1. Introduction

Oxidative stress mediated by reactive oxygen species (ROS) has been implicated as a major cause of cellular damage and contributes to inflammation, aging, cancer, arteriosclerosis, hypertension and diabetes [1–3]. Persistent ROS elevation is a result of an imbalance between ROS production and scavenging by endogenous antioxidants that directly or indirectly disturb physiological functions of many cellular macromolecules, such as DNA, proteins and lipids. Excessive ROS ultimately induce cell death, either by apoptosis or necrosis [4]. Mitochondrial dysfunction results in increased ROS production that enhances oxidative stress if the cellular defense systems are overwhelmed [5]. Previous studies have indicated that ROS might alter intracellular redox states, change the inner mitochondrial membrane potential (Δψ_m_) and release soluble inter-membrane proteins, including cytochrome c, from mitochondria into the cytosol [6,7]. It is also well known that ROS plays a crucial role in triggering the mitochondria-mediated apoptotic pathway, which is associated with activation of the caspase cascade and the family of Bcl-2 proteins [8–10].

**Figure 1:**
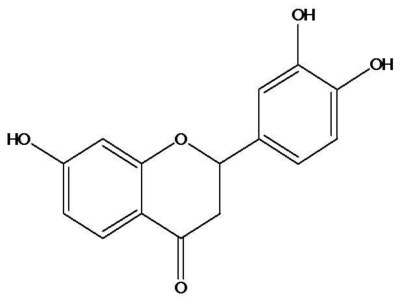
Chemical structure of butin (7,3′,4′-trihydroxydihydroflavone).

Flavonoids are a group of naturally occurring polyphenolic compounds found ubiquitously in fruits and vegetables, and represent substantial constituents of the non-energetic part of the human diet. Butin (7,3′,4′-trihydroxydihydroflavone, Figure 1), one of the most widely distributed flavonoids, is reported to be a potent antioxidant against oxidative stress-related diseases, such as cancer, aging, liver diseases and diabetes [11–14]. In previous reports, Zhang *et al.* demonstrated that butin protected cells against hydrogen peroxide (H_2_O_2_)-induced apoptosis by scavenging ROS and activating antioxidant enzymes [15], decreased oxidative stress-induced 8-hydroxy-2′-deoxyguanosine levels via activation of oxoguanine glycosylase 1 (OGG1) [16], and reduced oxidative stress-induced mitochondrial dysfunction via scavenging of ROS [17]. Considering mitochondria, the intracellular organelles producing the largest amount of ROS in cells, play a major role in the development of oxidative stress under both physiological and pathological conditions [18,19], mitochondrial dysfunction is most likely to be responsible for oxidative stress-induced apoptosis [20]. To extend our previous investigations, we focused on the effect of butin on mitochondria-mediated caspases dependent apoptotic pathway which is induced by oxidative stress in this study.

## 2. Results and Discussion

### 2.1. Effect of Butin on H_2_O_2_-Induced Δψ_m_ Depolarization

**Figure 2:**
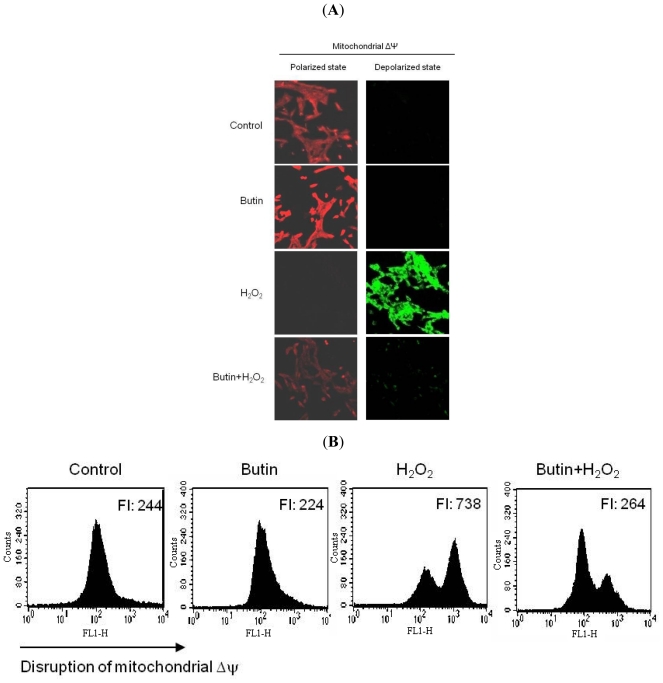
Effects of butin on H_2_O_2_-induced Δψ_m_ depolarization. Δψ_m_ was analyzed by (**A**) confocal microscope and (**B**) flow cytometer after staining cells with JC-1. FI indicated the fluorescence intensity of JC-1.

In a previous report, we have indicated that butin protected against H_2_O_2_-induced apoptosis [15]. Change in Δψ_m_ was examined to improve understanding of butin’s protection mechanism for H_2_O_2_-induced apoptotic process in terms of mitochondrial involvement. JC-1 is a cationic dye that indicates mitochondrial polarization by shifting its fluorescence emission from green (~525 nm) to red (~590 nm). As shown in Figure 2A, control cells and butin-treated cells exhibited strong red fluorescence (JC-1 aggregated form, indicative of mitochondrial polarization) in the mitochondria. However, H_2_O_2_ resulted in reducing red fluorescence and increasing green fluorescence (JC-1 monomer form, indicative of mitochondrial depolarization) in the mitochondria. Butin treatment blocked reducing red fluorescence and increasing green fluorescence in H_2_O_2_-treated cells. Image analysis data was consistent with flow cytometric data; the level of Δψ_m_ loss was increased in H_2_O_2_-treated cells, as substantiated by an increase in fluorescence with JC-1 dye. However, butin recovered the level of Δψ_m_ loss (Figure 2B), suggesting that butin partially inhibited loss of Δψ_m_ in response to H_2_O_2_ treatment.

### 2.2. Effect of Butin against H_2_O_2_-Induced Apoptosis

**Figure 3:**
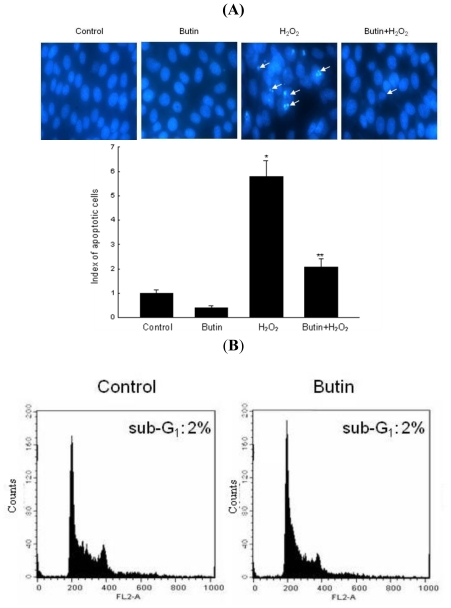

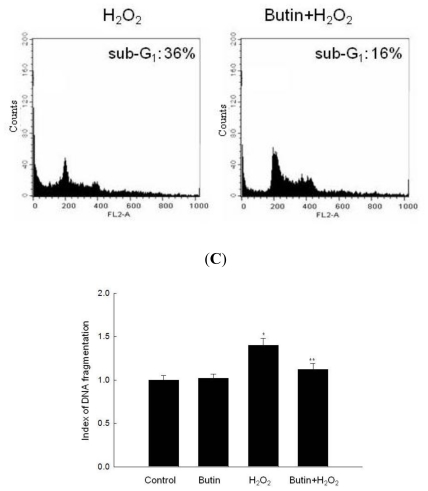
Effects of butin on H_2_O_2_-induced apoptosis. (**A**) Apoptotic body formation was observed under a fluorescence microscope and quantitated after Hoechst 33342 staining. Arrows indicate apoptotic bodies; (**B**) The apoptotic sub-G_1_ DNA content was detected by a flow cytometry after propidium iodide staining; (**C**) DNA fragmentation was quantified by ELISA kit. * Significantly different from control cells (*p* < 0.05). ** Significantly different from H_2_O_2_-treated cells (*p* < 0.05). *N* = 3 and “*n*” indicates the number of repetitions.

In order to confirm the cytoprotective impact of butin on H_2_O_2_-induced apoptosis, cell nuclei were stained with Hoechst 33342 for visualization by microscopy. The microscopic images in Figure 3A demonstrate that the control cells had intact nuclei, whereas H_2_O_2_-treated cells showed significant nuclear fragmentation, a characteristic of apoptosis. However, butin-pretreated cells exhibited a dramatic decrease in nuclear fragmentation induced by H_2_O_2_ treatment. In addition to morphological evaluation, the protective effect of butin against apoptosis was also confirmed by apoptotic sub-G_1_ DNA analysis. As shown in Figure 3B, an analysis of DNA content in H_2_O_2_-treated cells revealed a 36% increase in the apoptotic sub-G_1_ DNA content. However, butin decreased the apoptotic sub-G_1_ DNA content to 16%. Furthermore, H_2_O_2_-treated cells increased the levels of cytoplasmic histone-associated DNA fragmentations as compared to control, and butin significantly decreased the level of DNA fragmentation (Figure 3C).

**Figure 4:**
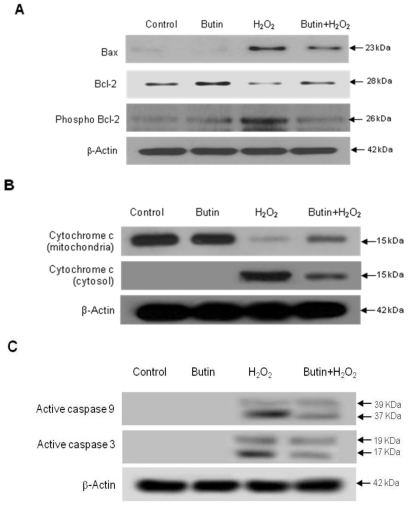
Effects of butin on mitochondrial apoptosis related proteins. Western blot analysis was performed. Cell lysates were electrophoresed and (**A**) Bax, Bcl-2, phospho Bcl-2; (**B**) cytochrome c; (**C**) active caspase 9, and active caspase 3 proteins were detected by their specific antibodies.

To further understand the protection mechanism of butin on H_2_O_2_-induced apoptotic process, we detected the protein expressions involved in mitochondria related apoptosis. Beforehand, changes in Bcl-2 expression, an anti-apoptotic protein, and Bax expression, a pro-apoptotic protein, were examined. As shown in Figure 4A, butin showed an increase in Bcl-2 expression and a decrease in Bax expression in H_2_O_2_-treated cells. It has been reported that Bcl-2 fails to inhibit cell apoptosis when inactivated via phosphorylation [9]. We noticed that butin also decreased phosphorylation of Bcl-2 (Ser 87) induced by H_2_O_2_ treatment. During the apoptotic process, Bcl-2 prevented the opening of the mitochondrial membrane pore, whereas Bax induced the opening of membrane pore [21]. Pore opening induces loss of Δψ_m_, which in turn induces the release of cytochrome c from the mitochondria [22]. As shown in Figure 4B, butin inhibited the release of mitochondrial cytochrome c. Next, caspase 9 activity was examined by Western blot since it is known that this enzyme is activated due to mitochondrial membrane disruption [23]. As shown in Figure 3C, treatment of cells with butin inhibited H_2_O_2_-induced active form of caspase 9 (39 and 37 kDa) and caspase 3 (19 and 17 kDa), a target of caspase 9. These results suggest that butin protects cells from apoptosis by inhibiting the caspase dependent pathway via mitochondria.

### 2.3. Effect of Butin on the SEK1-JNK-AP-1 Signaling Pathway

**Figure 5:**
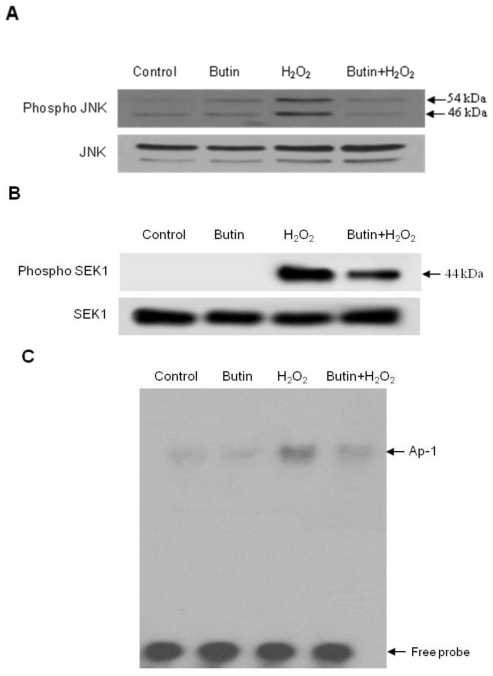

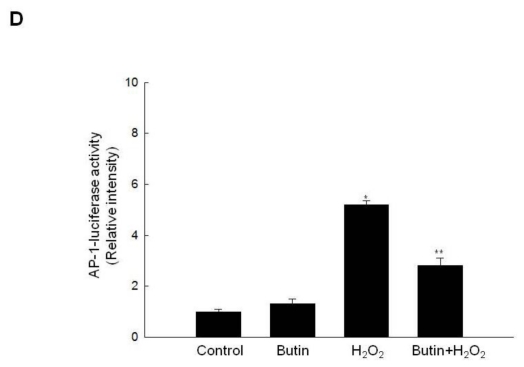
Effects of butin on H_2_O_2_-induced SEK1-JNK-AP-1 activation. Cell lysates were electrophoresed and the cell lysates were immunoblotted using (**A**) anti-JNK, phospho JNK and (**B**) -phospho SEK1 and -SEK1 antibodies; (**C**) AP-1 specific oligonucleotide-protein complexes were detected by the electrophoresis mobility shift assay; (**D**) The transcriptional activity of AP-1 was assessed using plasmid containing an AP-1 binding site-luciferase construct. * Significantly different from control (*p* < 0.05) and ** significantly different from H_2_O_2_-treated cells (*p* < 0.05). *N* = 3 and “*n*” indicates the number of repetitions.

The JNK signal pathway plays an important role in oxidative stress-induced apoptosis [24] and JNK translocates to the mitochondrial, then phosphorylates Bcl-2, and presumably inactivates them [25]. In addition, JNK induces the mitochondrial pathway of apoptosis by activating Bax [26], thus we tested whether butin regulates this signaling pathway. As shown in Figure 5A, butin inhibited JNK activation in H_2_O_2_-treated cells at 12 h. Moreover, SEK1 is known to be an upstream component in the JNK signaling pathway [27].

To investigate whether this upstream kinase plays a role in H_2_O_2_-induced JNK activation, SEK1 phosphorylation was determined by Western blot analysis. As shown in Figure 5B, SEK1 phosphorylation levels were increased in H_2_O_2_-treated cells at 6 h. However, treatment of cells with butin inhibited H_2_O_2_-induced SEK1 phosphorylation. AP-1 is a downstream target of the phospho JNK pathway, and activated AP-1 is involved in cell death including apoptosis [28]. Subsequently, we examined the effect of butin pretreatment on the DNA binding activity of AP-1 after H_2_O_2_ treatment at 24 h. As shown in Figure 5C, AP-1 DNA binding activity was increased in H_2_O_2_ treated cells, whereas treatment of cells with butin inhibited AP-1 activity.

The transcriptional activity of AP-1 was also assessed using a promoter construct containing AP-1 binding DNA consensus sequences, which were linked to a luciferase reporter gene. As shown in Figure 5D, butin inhibited the transcriptional activity of AP-1 induced by H_2_O_2_. These results suggest that butin inhibits H_2_O_2_-induced apoptosis via suppression of the SEK1-JNK-AP-1 pathway.

## 3. Experimental Section

### 3.1. Reagents

Butin was purchased from Wako Pure Chemical Ind., Ltd. (Tokyo, Japan). 5,5′,6,6′-Tetrachloro- 1,1′,3,3′-tetraethyl-benzimidazolylcarbocyanine iodide (JC-1) was purchased from Invitrogen (Carlsbad, CA, USA). The primary anti-B-cell lymphoma 2 (Bcl-2), -Bcl-2-associated x protein (Bax), -phospho Bcl-2, and -cytochrome c antibodies were purchased from Santa Cruz Biotechnology Inc. (Santa Cruz, CA, USA). Primary anti-caspase 9, -caspase 3, -c-Jun N-terminal kinases (JNK), -phospho JNK, -mitogen-activated protein kinase kinase-4 (SEK1), and -phospho SEK1 antibodies were purchased from Cell Signaling Technology (Beverly, MA, USA). A plasmid containing activator protein-1 (AP-1) binding site-luciferase construct was a generous gift from Professor Young Joon Surh of Seoul National University (Seoul, Korea). Propidium iodide and Hoechst 33342 were purchased from the Sigma Chemical Company (St. Louis, MO, USA).

### 3.2. Cell Culture

Chinese hamster lung fibroblasts (V79-4 cells) from the American type culture collection were maintained at 37 °C in an incubator, with a humidified atmosphere of 5% CO_2_ and cultured in Dulbecco’s modified Eagle’s medium containing 10% heat-inactivated fetal calf serum, streptomycin (100 μg/mL) and penicillin (100 units/mL).

### 3.3. Mitochondrial Membrane Potential (Δψ_m_) Analysis

Δψ_m_ analysis was determined by confocal image analysis and flow cytometer. The V79-4 cells were seeded at a concentration of 1 × 10^5^ cells/mL, and 16 h after plating, were treated with butin at 10 μg/mL, and after 1 h, 1 mM of H_2_O_2_ was added to the plate, and the mixture was incubated for 12 h. Cells were then harvested, and after changing the media, JC-1 was added to each well and was incubated for an additional 30 min at 37 °C. After washing with PBS, the stained cells were mounted onto microscope slide in mounting medium (DAKO, Carpinteria, CA, USA). Microscopic images were collected using the Laser Scanning Microscope 5 PASCAL program (Carl Zeiss, Jena, Germany) on confocal microscope [29]. In addition, Δψ_m_ analysis was also determined by flow cytometer. The cells were harvested, washed and suspended in phosphate buffered saline (PBS) containing JC-1 (10 μg/mL). After incubation for 15 min at 37 °C, the cells were washed and were suspended in PBS and were analyzed by flow cytometer [30].

### 3.4. Western Blot Analysis

Cells were seeded at a concentration of 1.5 × 10^5^ cells/mL and 16 h after plating, cells were treated with butin at 10 μg/mL, and after 1 h, 1 mM of H_2_O_2_ was added. After 6, 12 or 24 h, cells were harvested, washed twice with PBS, lysed on ice for 30 min in 100 μL of a lysis buffer (120 mM NaCl, 40 mM Tris (pH 8), 0.1% NP 40) and then centrifuged at 13,000 × g for 15 min. The supernatants were collected from the lysates and the protein concentrations determined. Aliquots of the lysates (40 μg of protein) were boiled for 5 min and electrophoresed in 10% sodium dodecysulfate-polyacrylamide gel. The blots in the gels were transferred onto nitrocellulose membranes (Bio-Rad, Hercules, CA, USA), which were then incubated with the primary antibodies. The membranes were further incubated with the secondary immunoglobulin-G-horseradish peroxidase conjugates (Pierce, Rockford, IL, USA). Protein bands were detected using an enhanced chemiluminescence Western blotting detection kit (Amersham, Little Chalfont, Buckinghamshire, UK), and then exposed onto X-ray film.

### 3.5. Nuclear Staining with Hoechst 33342

Cells were seeded at a concentration of 1 × 10^5^ cells/mL, and 16 h after plating, were treated with butin at 10 μg/mL. After 1 h, 1 mM of H_2_O_2_ was added to the plate and the mixture was incubated for 24 h. 1.5 μL of Hoechst 33342 (stock 10 mg/mL), a DNA specific fluorescent dye, was added to each well and incubated for 10 min at 37 °C. The stained cells were then observed under a fluorescent microscope, which was equipped with a CoolSNAP-Pro color digital camera, in order to examine the degree of nuclear condensation. The percentage of apoptotic cells (apoptotic index) was assessed by counting 3 random fields in triplicate wells.

### 3.6. Detection of Apoptotic Sub-G_1_ Hypodiploid Cells

The amount of apoptotic sub-G_1_ hypodiploid cells was determined using flow cytometer [31]. Cells were seeded at a six-well plate at a concentration of 1 × 10^5^ cells/mL, and 16 h after plating, were treated with butin at 10 μg/mL. After 1 h, 1 mM of H_2_O_2_ was added to the plate and the mixture was incubated for 24 h. Cells were harvested and fixed in 1 mL of 70% ethanol for 30 min at 4 °C. The cells were then washed twice with PBS, and incubated for 30 min in the dark at 37 °C in 1 mL of PBS containing 100 μg of propidium iodide and 100 μg of RNase A. A flow cytometric analysis was performed using a FACS Calibur flow cytometer. Sub-G_1_ hypodiploid cells were assessed based on histograms generated by the Cell Quest and Mod-Fit computer programs.

### 3.7. DNA Fragmentation

Cells were seeded at a concentration of 5 × 10^4^ cells/mL, and 16 h after plating, cells were treated with butin at 10 μg/mL. After 1 h, 1 mM of H_2_O_2_ was added to the plate and the mixture was incubated for 24 h. Cellular DNA-fragmentation was assessed by analyzing cytoplasmic histone-associated DNA fragmentation, using a kit from Roche Diagnostics according to the manufacturer’s protocol.

### 3.8. Preparation of the Nuclear Extract and Electrophoretic Mobility Shift Assay

Cells were seeded at a concentration of 1.5 × 10^5^ cells/mL, and 16 h after plating, cells were treated with butin at 10 μg/mL. After 1 h, 1 mM of H_2_O_2_ was added to the plate and the mixture was incubated for 24 h. After 24 h, cells were harvested, and subsequently lysed on ice with 1 mL of lysis buffer (10 mM Tris-HCl, pH 7.9, 10 mM NaCl, 3 mM MgCl_2,_ and 1% NP-40) for 4 min. After 10 min of centrifugation at 3000 × g, the pellets were re-suspended in 50 μL of extraction buffer (20 mM HEPES, pH 7.9, 20% glycerol, 1.5 mM MgCl_2_, 0.2 mM EDTA, 1 mM DTT, and 1 mM PMSF), incubated on ice for 30 min and centrifuged at 13,000 × g for 5 min. The supernatant (nuclear protein) was stored at −70 °C after determining the protein concentration. Oligonucleotides containing transcription factor AP-1 consensus sequence (5′-CGC TTG ATG ACT CAG CCG GAA-3′) were annealed, labeled with [γ-^32^P] ATP using T4 polynucleotide kinase and used as probes. The probes (50,000 cpm) were incubated with 6 μg of the nuclear extracts at 4 °C for 30 min, to reach a final volume of 20 μL, containing 12.5% glycerol, 12.5 mM HEPES (pH 7.9), 4 mM Tris-HCl (pH 7.9), 60 mM KCl, 1 mM EDTA, and 1 mM DTT with 1 μg of poly (dI-dC). The binding products were resolved on 5% polyacrylamide gel and the bands were visualized by autoradiography.

### 3.9. Transient Transfection and AP-1 Luciferase Assay

Cells were seeded at a concentration of 1.0 × 10^5^ cells/mL, and 16 h after plating, cells were transiently transfected with plasmid harboring the AP-1 promoter using DOTAP as the transfection reagent, according to the manufacturer’s protocol (Roche Diagnostics, Portland, OR, USA). Following overnight transfection, cells were treated with 10 μg/mL of butin, and after 1 h, 1 mM of H_2_O_2_ was added to the plate for 24 h. Cells were washed twice with PBS and lysed with reporter lysis buffer (Promega, Madison, WI, USA). Following vortex mixing and centrifugation at 12,000 × g for 1 min at 4 °C, the supernatant was stored at −70 °C for the luciferase assay. After mixing 20 μL of cell extract with 100 μL of luciferase assay reagent at room temperature, the mixture was placed in an illuminometer to measure the light produced.

### 3.10. Statistical Analysis

All measurements were performed in triplicate and all values were represented as the mean ± standard error of the mean (SEM). The results were subjected to an analysis of variance (ANOVA) using the Tukey’s test to analyze difference. *P* < 0.05 were considered statistically significant.

## 4. Conclusions

In this study, treatment of cells with H_2_O_2_ resulted in significant collapse of Δψ_m_, however, treatment with butin recovered H_2_O_2_-induced depolarization of Δψ_m_. In addition, H_2_O_2_ treatment induced a dramatical increase in Bax expression and decrease in Bcl-2 expression, suggesting that changes in the pro-apoptotic and anti-apoptotic Bcl-2 family proteins may contribute to apoptosis. Moreover, elevation of phospho Bcl-2 by H_2_O_2_ treatment further helps to reduce its ability to bind with Bax and enhance translocation of Bax from the cytosol to mitochondria, leading to an enhanced susceptibility of the cells to apoptosis [32]. Butin significantly restored these changes induced by H_2_O_2_. These results confirmed that butin inhibited H_2_O_2_-induced apoptosis associated with regulation of Bcl-2 family proteins. Changes in caspases 9 and 3 protein expressions were evaluated for the underlying mechanisms, as cleaved caspases 9 and 3 represent downstream signals of apoptosis and the Bcl-2 protein can prevent activation of caspases during apoptosis. Butin inhibited H_2_O_2_-induced activation of caspases 9 and 3. Treatment of cells with butin showed anti-apoptotic effects in cells exposed to H_2_O_2_, as shown by apoptotic body formation, sub G_1_-hypodiploid cells levels and nuclear fragmentation.

Various studies have suggested possible mechanisms for the JNK pathway also relate to mitochondrial depolarization and apoptosis induction. It has been reported that JNK translocates to the mitochondria, then phosphorylates Bcl-2 and Bcl-XL, anti-apoptotic members of Bcl-2 family, and presumably inactivates them [26]. In addition, SEK1-JNK-AP-1 activation has been suggested as a critical component in the oxidative stress-induced apoptosis process [33]. Butin inhibited H_2_O_2_-induced JNK phosphorylation, resulting in a decrease of AP-1 activity. H_2_O_2_-induced phosphorylation of SEK1, an upstream regulator of JNK, was also attenuated by butin treatment. These results demonstrated that butin attenuated H_2_O_2_-induced apoptosis through the SEK1-JNK-AP-1 pathway. Our previous study has demonstrated that PI3K-Akt pathway also involved in cytoprotective effect of butin against oxidative stress-induced cell damage. Butin induced OGG1, DNA base repair enzyme, via regulation of PI3K-Akt pathway [16].

The structural requirement for effective radical scavenging criteria in flavonoids is the presence of a 3′,4′-orthodihydroxy group (catechol structure) in the B ring or in the A ring, and the C2–C3 double bond conjugated with a carbonyl group in the C ring. The existence of C2–C3 double bond in the C ring is important for electron delocalization from the B ring, enhancing radical-scavenging capacity [33]. The unique feature of butin as compared to flavonoid is partially consistent with these criteria as mentioned above. The absence of C2–C3 double bond in the C ring of butin might be expected to have weak antioxidant activity. Nevertheless, butin increased antioxidant activity via radical scavenging activities and enhancing the effects of antioxidant enzymes [15]. Comparing the structural criteria with radical scavenging activity among flavonoids, including butin, remains a subject for further study. To the best of our knowledge, the exact cellular mechanism of butin on cells has not been well understood. Thus in the present study, we focused on butin effects on mitochondria-dependent apoptosis induced by oxidative stress and it is the first report on cytoprotective mechanisms of butin. Many of different clinical mechanisms of flavonoids have been related with their antioxidant properties, either through their reducing capacities or influences on intracellular components. The precise mechanisms by which flavonoids exert their beneficial or toxic actions remain unclear [34,35]. Although Maruta *et al*. reported that quercetin and kaempferol were mutagenic to hamster fibroblasts [36], however there is increasing interest in research on flavonoids, due to growing evidence of their health benefits through epidemiological studies. We have reported that morin (2′,3,4′,5,7-pentahydroxy-flavone) protected against oxidative stress-induced cellular damage in lung fibroblast cells [37,38]. In addition, myricetin (3,3′,4′5,5′,7-hexahydroxylflavone) prevented cells from oxidative stress-induced apoptosis via regulation of PI3K/Akt and MAPK signaling pathways in lung fibroblast cells [39]. In the present study, the evaluation of butin on lung protection induced by oxidative stress was not performed *in vivo*. However, baicalin (5,6,7-trihydroxyflavone), similar compound to butin, showed protective effect on lipopolysaccharide-induced lung damage in rats with administration of 20 mg/kg [40], and an *in vivo* study of butin and its underlying metabolism (absorption, distribution, metabolism, excretion) remains for further study.

Many flavonoids are shown to have antioxidant activity, coronary heart disease prevention, anti-inflammation, oestrogenic activity, anticancer activity, and other biological activities [41–43]. As such research progresses, potential application of flavonoids in either foods or pharmaceutical supplements will expand. Considering butin’s reduction of mitochondria-dependent apoptosis, it might give a hopeful picture for oxidative stress related diseases such as aging, diabetes, and neurological diseases for the initial step of clinical trial and development of raw materials of medicine. Accordingly, an appropriate system for assessment of intake of butin needs to be developed for further study.

**Figure 6:**
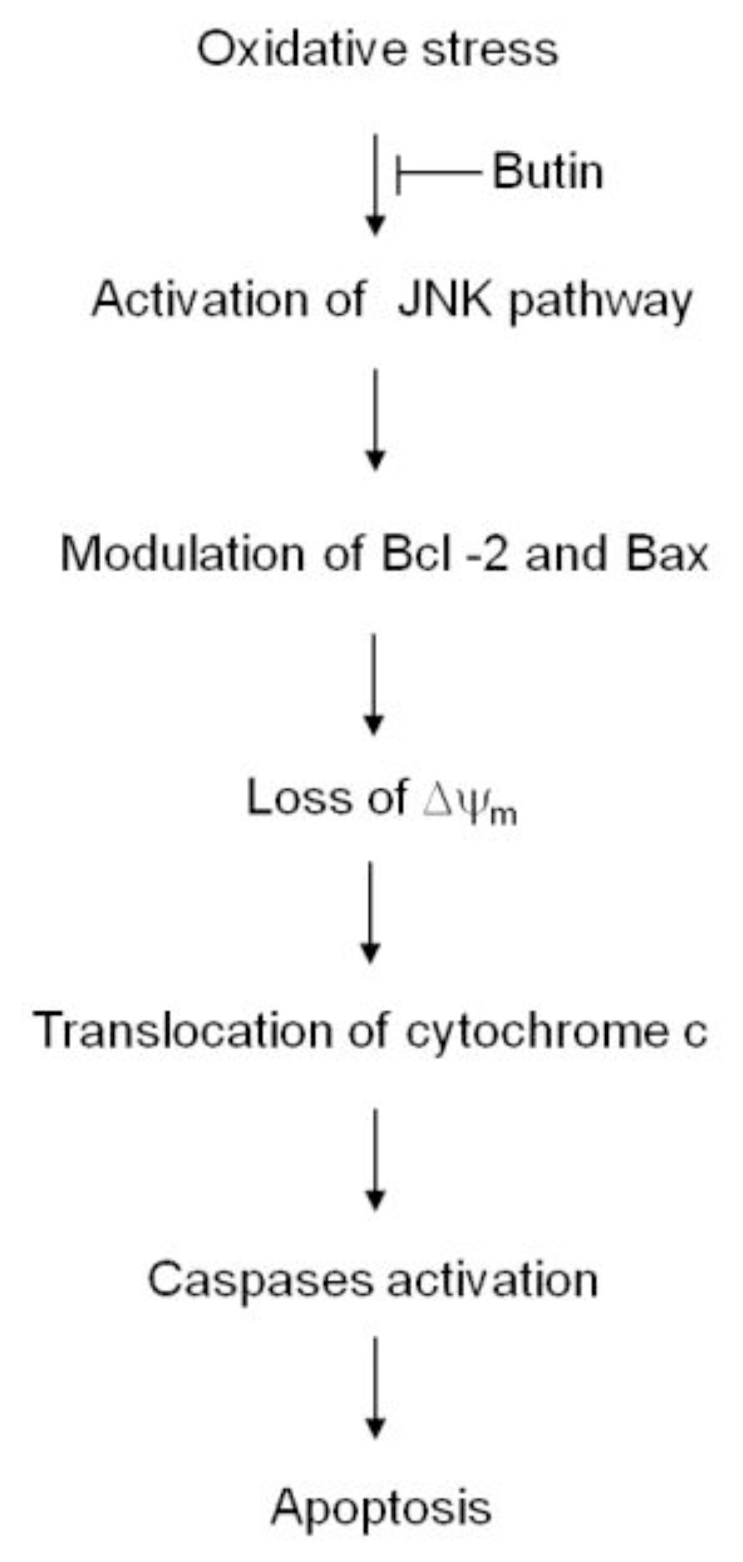
A proposed cyto-protective pathway of butin, which explains its properties against oxidative stress-induced mitochondrial involved apoptosis.

Taken together, the protective effect of butin against H_2_O_2_-induced apoptosis was exerted via blockage of membrane potential depolarization, inhibition of the JNK and mitochondria involved caspase-dependent apoptosis pathways. Therefore, we suggest that inhibition of these pathways by butin may provide oxidative stress protection (Figure 6).
